# Academic writing in medicine and healthcare

**DOI:** 10.3389/fmed.2025.1617752

**Published:** 2025-07-24

**Authors:** Airazat M. Kazaryan, Martin Hagve, Knut Magne Augestad, Tom Nordby, Bjørn Edwin, Waleed Ghanima

**Affiliations:** ^1^Department of Surgery, Østfold Hospital Trust, Grålum, Norway; ^2^Department of Gastrointestinal and Pediatric Surgery, Oslo University Hospital - Ullevål, Oslo, Norway; ^3^Department of Surgery N1, Yerevan State Medical University after M. Heratsi, Yerevan, Armenia; ^4^Department of Faculty Surgery N2, I.M.Sechenov First Moscow State Medical University, Moscow, Russia; ^5^Department of Gastrointestinal Surgery, University Hospital in Northern Norway, Tromsø, Norway; ^6^Research Group for Gastrointestinal Surgery, Medical Faculty, University of Tromsø – Norwegian Arctic University, Tromsø, Norway; ^7^Department of Gastrointestinal Surgery, Akershus University Hospital, Lørenskog, Norway; ^8^Institute for Clinical Medicine, University of Oslo, Oslo, Norway; ^9^Department of Surgery, Helgeland Hospital Trust, Sandessjøen, Norway; ^10^Department of Surgery, Columbia University, New York, NY, United States; ^11^Intervention Center, Oslo University Hospital - Rikshospitalet, Oslo, Norway; ^12^Section for Hepatopancreatobiliary Surgery, Oslo University Hospital - Rikshospitalet, Oslo, Norway; ^13^Department for Hematology and Oncology, Østfold Hospital Trust, Grålum, Norway; ^14^Department of Research, Østfold Hospital Trust, Grålum, Norway

**Keywords:** research, motives, practice, publication, recruitment, management

## Abstract

The article highlights factors that can motivate doctors to do medical research. Various types of medical publications are presented. In addition, prerequisites that can contribute to successful publication are described. An overview of trends in research publication is given as well as an explanation of the terms discovery and innovation. The role and importance of research and scientific writing for progress in medicine and healthcare are discussed here. The connection between medical practice and research is also highlighted. Not least, the need for support from supervisors and management in the research and publication process is highlighted.

## Introduction

Acquiring knowledge is an essential prerequisite for the practice of medicine. Continual learning of new information is crucial for improving and advancing patient care.

New knowledge in medicine is generated through research. This is a responsibility and an obligation, but also an opportunity for the medical profession and healthcare organizations to improve patient treatment and knowledge base for the best possible treatment. These aspects are also relevant in the context of the best possible resource allocation in public health.

A crucial aspect of doing comprehensive research is the capacity to communicate and disseminate study findings effectively. Hence, a strong correlation exists between research and communication proficiency.

## Statutory regulations, requirements for research understanding within specialist education

Regulations regarding research in health care vary worldwide. However, the role of medical research is pointed out in national legal healthcare regulations of vast majority of countries in the world. As an example, research is one of four statutory legal duties in the specialist healthcare services, alongside patient treatment, education of healthcare personnel, and training in Norway (1, 2). The Constitution of the World Health Organization (WHO) from 1948, highlights these aspects by the statements “… to promote co-operation among scientific and professional groups which contribute to the advancement of health,” “… to promote and conduct research in the field of health” (3). Furthermore, WHO states that the strategy in health care should be continuously adjusted by new knowledge coming from medical research (4).

The requirement for interpreting research, which includes insight into research methodology, analytical reading of medical research literature, and knowledge of relevant legislation and research ethics, is specified in the regulations for specialist education in many countries worldwide (5).

## Why should medical doctors perform academic research and publish?

There are several motivations behind the wish among doctors and healthcare providers to publish research articles (6).

### Egoistic motives

*1. Career*

Scientific publishing can strengthen the opportunities for career development and provide the opportunity to work in a field of your choice. Publishing research papers may allow for positioning oneself in a competitive professional environment. If you opt for a doctoral degree (Ph.D., i.e., Philosophy Doctor or equivalent), publications are normally and particularly in medical research fundamental to defending the thesis. Holding a doctoral degree is an advantage in job applications, particularly at university hospitals, and in some cases, it may be mandatory. Publications may also boost the possibilities for further research and access to research funding and positions.

*2. Recognition*

Many people feel a sense of satisfaction when they see their name in a journal and are happy to be cited by others and thus contribute to the development of medicine. Some want to contribute something significant - and to be remembered for it. To achieve this, the doctor needs to publish and document their discoveries and practice through research so that it is available to others. The historical recognition of Ernst Heller, who pioneered esophagomyotomy for achalasia, underscores the importance of publishing new techniques to ensure their adoption and legacy (7). Pioneers may not achieve recognition for discoveries that may rather be associated with researchers that have replicated or modified the pioneers’ developments and made the findings available through publishing.

*3. Expand the knowledge base.*

When you write an article, you deepen your knowledge and understanding of the topic in question, as well as the conceptual framework and research methods of different studies in general. In this way, you improve your knowledge to benefit the patients and yourself in your own professional work. It also strengthens the ability to interpret and understand other people’s research. In this way, you may be able to contribute to ensure that the treatment you provide has the best possible evidence. Furthermore, you position yourself better for academic and professional discussions (8).

*4. Professional collaboration and networking.*

The more you write and contribute to the research field, the more attractive you and your research group may be as a collaborative research partner. Collaboration on an international level expands the opportunities to do research on current hot spot topics as well as the opportunities to publish. It also provides a basis for further exchange of experiences and improvement of the quality of your clinical and scientific work. Such cooperation is of great value for the development, implementation, and quality assurance of new methods. Networking through collaboration may also allow access to groups and associations that may further boost your research (9).

*5. Economic motives.*

Most medical journals publish scientific articles without remuneration to the authors. Sometimes, the authors have to cover expenses related to the publication process (for example, for the publication of color illustrations or open-access journals). The research activity is not directly financially beneficial, but significant publications can strengthen the opportunities to contribute to research grants and funding and participation in scientific conferences. It can also lead to increased flexibility in the working situation. The financial incentive may be present for pharmaceutical research and research related to medical devices. There are strict ethical regulations in most countries for such relationships with commercial partners (10).

### Altruistic motives

The desire to share thoughts, experiences, and discoveries with others is undoubtedly an important reason for writing scientific articles. The main purpose of professional publishing is to spread knowledge and improve patient care and diagnostics (11). A doctor must feel obligated to publish important clinical observations, regardless of whether they describe the doctor’s achievements or his unfortunate experiences.

### Other motives

There is also a neutral motive for doing research as part of the medical profession: the desire to constantly discover and find something new without directly connecting with the above-stated motives (“curiosity”).

### How can doctors engage in academic activities and publish?

Doctors are not only practitioners of medicine; some are also teachers and researchers. The question is how to take care of such a triathlon function with increasing demands for quality, clinical competence, narrowing sub-specialization, technological development, and increasing pressure related to work tasks. Publishing scientific articles can be demanding, especially for highly specialized cross-disciplinary and complex topics. There are, nevertheless, many examples of excellent scientific works where valuable and complex information is expressed so simply and clearly that it is easily understood by readers who themselves are not specialists in the field.

“What can I write about?.” Medical scientific articles can be divided into three main types: case reports, original articles, and review articles (12). An interesting case report can be the first incentive to delve into the medical literature on the issue in question, give a presentation to colleagues, or write a case article for a medical journal. Many start their scientific careers with interesting case studies. However, many journals have either stopped publishing case reports or limited them to only very unique reports. At the same time, some journals have specialized in case reports.

Original articles are the locomotive in academic medicine and the primary type of scientific publication. Summarizing one’s own or the institution’s experience with treatment or diagnostics in a retrospective analysis may be an alternative. Unlike true medical research studies, these are often quality assurance, “association,” or “observational” studies that may fall outside the Ethical Committee’s mandate. These studies may generate hypotheses and indicate topics for further research. The interface between what constitutes true research and quality assurance can be challenging, and counseling with an Ethical Committee may be advised. Prospective and particularly randomized controlled studies provide a higher level of research evidence but involve considerably more resource use and research methodological expertise. Original articles may also be based on experimental studies. These research studies allow for the more targeted determination or control of certain properties of the setup and provide good opportunities to test the hypotheses that are deducted based on theoretical considerations. The study findings may, however, not be translatable to clinical practice.

An review article typically reflects the latest trends and “state-of-the-art” innovations related to the topic being addressed. The proportion of classic (recently called descriptive) review articles has decreased in recent years. Now, these are primarily written by well-known experts within the specific issue, often in the form of invited articles. Meta-analyses and systematic reviews are becoming the dominant type of review article and require thorough literature searches and skills for literature quality and assembled analyses. A meta-analysis of randomized studies provides a summary of existing evidence and is characterized as the highest level of evidence.

There are other types of articles, such as commentaries, editorials, research letters, and short reports. They present concise posts and can express personal opinions without necessarily having a strong research background. Moreover, they can be published without peer review, but only at the editor’s decision. Such articles open up reflections that can stimulate further research.

## Recruitment and research opportunities for doctors

Ways to recruit and stimulate doctors to do research vary around the world but can be challenging. Research is often less well paid than clinical work, and research work is often not prioritized in practical clinical everyday life. Recruitment of doctors to research can thus be challenged both at institutions that are affiliated and not affiliated with a university (13). For clinicians, it may be more important to position themself clinically than in terms of research. Such a low priority could affect the institutional possibilities of following evidence-based medicine in daily practice (14–16).

At the university hospitals, research can be carried out within the framework of university academic positions such as full professor, associate or assistant professor, lecturer, and research fellow. These positions also usually involve teaching. The senior academic positions in clinical medicine are usually arrangements combined with consultant positions at the associated hospital.

There are goal-oriented Ph.D-fellow positions based on granted research scholarship. The advantage of the position is that the research fellow can often devote all of his/her time to research. The disadvantage is the absence from clinical activity and a reduction in salary compared to clinical positions. This explains why many consider these positions not suitable for themselves. In addition, these positions are often based on a grant allocation for a specific research project. The competition for research funds is tough, so several potentially good research projects do not receive funding via the scholarship scheme and are not realized.

There are special clinical positions in some countries which imply some time allocated to research. The doctor retains a salary corresponding to his clinical competency/stage in specialization, and he/she continues to develop his clinical competence. There are significant variations in how the combined positions are practiced, regulated, and financed.

Besides the dedication of working time to research, it is a well-known fact that many doctors use their private unpaid time to do research. Type or form of dedication to research activity within the context of clinical medicine will vary across medical specialties, and also by where the doctor is in the career.

## Science and clinical practice

Many doctors are good clinicians and do not necessarily need to publish scientific articles to achieve high competence, recognition, status, or privileges. However, some want to contribute scientifically and should be given the opportunity to do so within the framework of responsible clinical duties. A doctor’s involvement in research will promote both the person’s own but also the group’s knowledge and professional development. A challenge for some is that clinical work is often more easily visible and rewarded in everyday life than research, which may be more difficult to spot in the clinic, particularly the work leading up to the final paper.

All doctors must and should have a certain relationship with research, even if one does not necessarily need to do research actively throughout their career. This requires that research should be the focus during education at medical school and in all hospitals. It is important that doctors who enter into research projects and who assume responsibility for its execution have arranged clinical duties in a way that enables successful project processing, both by means of sufficient time frame, resources and patients treatment flow. Help from leadership plays an essential role here.

Research experience assists in a more creative way of thinking and a more evidence-based practice. It can contribute to better clinical decisions and easier knowledge acquisition (17).

## Facilitation and support by colleagues and leadership

Doctors who want to start research should get support from senior colleagues and established researchers to develop scientific critical thinking. This will contribute to the creation of conditions for potential research professional growth so that doctors can achieve the ability to generate their own original ideas, independently formulate research problems, and solve them. In addition to guidance, it is important to give junior researchers the opportunity to express their own ideas and opinions and actively argue for them. The role of administrative managers is also important. Junior doctors with academic ambitions must receive support from the management both in the form of encouragement for research and efforts must be made to adapt a work plan so that doctors who are interested have the opportunity to do research.

## Discoveries, innovation/invention, and research

Both discovery and innovation create new knowledge, but these terms are not the same (18). When Flemming observed the effect on bacteria in a patch of fungus in a forgotten Petri dish, it was an accidental discovery. Afterward, several studies tested and further developed this discovery. Surely, one should have solid knowledge and analytical abilities to further develop discoveries for practical use and benefit. Carrying out a doctoral project contributes to the systematic rebuilding of the doctor’s research competence and acceleration of further productivity in research work.

Innovation can be based on something already existing but shows new ways of using existing substrates or methodologies. For example, keyhole surgery for removing the gallbladder is regarded as a significant innovation that further led to the development of laparoscopic techniques in gastrointestinal surgery. It is also worth noting that the methods of open cholecystectomy and laparoscopy were practiced decades earlier. The combination of these two methods, together with the introduction of image projection on a screen, led to innovation in the form of laparoscopic cholecystectomy.

However, there was not adequate evidence to support this method as safe and effective until several years after the innovation was widely put into use. Nevertheless, a number of smaller pilot studies were published before proceeding with studies with a high level of evidence. This shows how implementing discoveries for use in routine practice can be time-consuming and challenging. Several early pioneers in laparoscopic surgery experienced a long period of distrust and opposition from colleagues in the early stage of introducing new treatment methods. The pioneers even risked being suspended from the right to treat patients (19). It can be really demanding to get acceptance for pioneering work and to have it published. Pioneering work in clinical activities also entails a risk of unexpected outcomes, which must be taken into account in planning, implementation, and patient management.

Chance can play an important role in innovation and medical progress. The discovery of ciclosporin, which has dramatically changed transplant surgery, occurred as a surprising incidental finding during the examination of soil samples from Norway with the intention of finding antifungal substances. Surprisingly, an immunosuppressive effect of the samples was discovered (20). This led to the discovery of ciclosporin, which has radically changed transplant surgery. A Nobel prize laureate Jacques Monod wrote in his article “On Chance and Necessity,” “… an event, totally fortuitous, coming with a pure chance may enter into the field of necessity with an implacable certitude” (21).

This shows how discoveries and innovations, together with a good portion of coincidences within research, can fundamentally change our clinical everyday life. This should not, however, disregard the importance of more routine research that ensures quality and further develops new methods in diagnostics and treatment, as well as popularizes new knowledge and spreads it around the world (22, 23). The methods can also be further developed by both clinical experience and innovations in other areas, for example, better video processors and image chips in laparoscopy and endoscopy, based on technology development for other purposes. This leads to significant improvements in safety, efficiency, and results compared to those seen just a few years ago (24, 25).

Specific guidelines have been established to assist in developing innovations, from idea/innovation to pilot series and, finally, to randomized controlled trials. For example, the IDEAL (Idea, Development, Exploration, Assessment, Long-term study) guidelines gained wide acceptance within surgery and interventional treatment (26).

## Supervision and planning

There are two main ways to guide a researcher. One is when a supervisor, usually a colleague with research experience, guides a young researcher. In addition, a researcher can be guided through the peer review process by reading criticism from peer reviewers and editors (27). Many more congress abstracts are published than original articles. This partly indicates how difficult it is to publish and how difficult it can be to get through the selection peer-reviewing process in scientific medical journals. Rejection of manuscripts and then submission to another journal is common.

Naturally, the essence matters in the writing process. A well-planned study will have no scientific value without a good and concrete problem and proper material. A research protocol with a well-thought-out problem and methods will facilitate work. Originality, i.e., new contributions to existing literature and knowledge, is a strength in the publishing process, but articles that substantiate previous findings are also significant contributions.

## Permits and reporting

In addition to the research work itself, “administrative” research activity includes ethical approvals, approvals for use and registration of patient data, institutional approvals, clinical trial agreements with collaborators, and registration in different recognized national and international trial registries. Patient consent is often required in clinical research as a prerequisite for starting research data collection. There is a requirement to complete a course in animal welfare in the case of experimental studies on animals.

In recent years, many journals have required the presentation of articles according to prepared standardized guidelines for specific types of articles. Examples are CONSORT (Consolidated Standards of Reporting Trials) for randomized studies, PRISMA (Preferred Reporting Items for Systematic Reviews and Meta-Analyses) for systematic reviews or meta-analyses, and STROBE (Strengthening the Reporting of Observational Studies in Epidemiology) for observational studies (28, 29).

Today, it is common to publish the protocols from randomized trials in international registries of clinical trials prior to commencement or early after commencement. An example of this is clinicatrial.gov. Publication in ClinicalTrials.gov (administrated by the U.S. National Library of Medicine) or the Clinical Trials Information System (administrated by the European Medical Agency) before starting a randomized controlled trial is also a condition for publishing in most recognized journals. They are now also subject to stricter requirements for the publication of any conflicts of interest (30) and for specifying what each co-author has contributed in accordance with the Vancouver publication regulations (31, 32).

All these “administrative” activities take time to implement. At the same time, this additional documentation helps to ensure better quality in the research. This reflects a tendency toward demands and expectations in society regarding transparency and consideration of potential conflicts of interest. Some institutions offer professional help for these aspects of the research activities. All these parts of the research are essential not only for being able to perform the research but also for being able to adhere to institutional and national guidelines for research, which is the researcher’s responsibility.

There are several online tools that allow multi-functional research profiles, including research search engines, tracking of research articles, and access to an international network of researchers working in the same or similar research field (ORCID; Google Scholar; Scopus Author ID; Researchgate; Web of Science ResearcherID; Publons). These provide the opportunity to measure research indices, including the H-index (Hirsch index). These new registration tools simplify the researcher’s work. ORCID (Open Researcher and Contributor ID) stands out in that it provides a unique ID number to researchers that is accepted by most serious scientific journals. This is a universal ID that simplifies communication between researchers and journals, both when the researcher is in the role of author and peer reviewer (33). Several countries also have national research tools, which, among other things, create research profiles for individual researchers. For example, in Norway, there is CRIStin (Current Research Information SysTem In Norway) - a national system for research documentation administered by the University of Oslo (34).

## Where to publish?

You have written an article and must find the right journal to publish your findings (27). Important considerations for choosing an appropriate journal are: (1) achieving a wide range of readers; (2) achieving a proper readership; (3) publishing in a good-rated and respected journal; (4) minimizing the possibility of rejection; (5) short processing time; (6) low article processing charges.

Unfortunately, these goals cannot always be achieved simultaneously (35). A sober and objective attitude to the value of the work is important. One can create a kind of algorithm to find a suitable journal. First of all, it is important that the authors examine the guidelines for publication in the relevant journal and also what types of articles are preferred. Most medical journals traditionally prefer original clinical or experimental studies, and only a few are focused on review articles and case reports. It turns out that many journals actually increase their share of review articles since these generate more citations. This can perhaps partly be explained by the fact that many researchers find it easier to refer to an overview article than to look for all the original articles to support a claim. Submission of a manuscript to a journal that does not match will result in automatic rejection. Checking published articles in the latest issues of the journal and whether the content, pattern and style of your own article are in accordance with the articles that have been published can be time-saving in the process of finding a suitable journal. If your article can solve the problem discussed in a previous article in a journal, it’s a good idea to submit the article there. Choosing the right journal is an important step. It can be helpful to ask for advice from senior colleagues who have extensive experience in own accepted and rejected manuscripts. Journals indexed in appropriate channels like PubMed and Scopus, are typically preferred and may ensure wide recognition of the research. Not indexed open access journals are often not serious and better be avoided.

There is a tendency toward increasing demands for evidence in scientific articles. Although medicine is referred to as a “hard science” (“hard/exact science”), it is “softer” than the classic “hard” scientific disciplines such as mathematics, physics, and chemistry. As part of this, the editors of medical journals strive for strict statistical analysis, advanced statistics, and randomized multicenter studies (36).

## Assessment of research quality

Scoring of research quality is essential to be able to control and prioritize research efforts both at international, national, and institutional levels. It is natural to think that what is mentioned often (cited) is important and deserves mention. Despite the weaknesses in this practice, it is widely accepted that ranking based on the number of citations, i.e., the Impact factor for journals and the Hirsch index (h-index) for individual researchers, plays an important role (37, 38). Some of the problems with this are related to the fact that publications from a long time ago receive lower citation rates, even though they may often have had great innovative significance. This applies especially after the digitization of research publishing, which has meant that the number of both publications and citations has increased dramatically. Therefore, a fair scoring of research carried out in different eras according to the citation principle is very demanding and almost impossible.

The citation tradition also differs within different professional fields. For example, articles in physics apply significantly fewer citations in a reference list than in medicine. Weighing research quality will also give an unnaturally high number of citations to review articles, as these are often easier to cite than the original work. In addition, the system inclines both researchers and journals to cite their own works in order to increase their own citation index. The citation rate is lacking in relation to studies carried out within secret research projects, usually linked to national security. These studies could have had great significance for humanity. For example, this applies to studies in nuclear physics. However, these have either never been openly published or they have been published decades after being performed. Thus, many of these secret researchers are not credited for their outstanding work or credited considerably time-lagged (39).

In recent years, several other bibliometric scoring tools have gained increasing use, among them SCImago journal Rank, which is citation-based, as well as several alternative non-citation-based so-called altmetric tools (40, 41). These tools calculate research impact based on multiple online exposures, for example in social media, online news media, online reference managers, etc. PlumX metrics is the most widespread almetric tool in the world.

## Open access and “preprint repositories”

Since the 1990s, some journals have offered so-called open access to articles, although this only became common in the early 2000s (42, 43). BioMed Central was one of the first publishers to offer such an option. These journals established a different model for publications – free access via the internet for readers of articles, but the author or the author’s institution must pay for publication in the journal. Despite some initial skepticism, the vast majority of journals have now gradually introduced the possibility of open access (Open Access) to their articles. Several journals have established an additional version of the journal with alternative open access as a publishing platform, such as BMJ Open, BJS Open, and Ann Surg Open. Numerous academic institutions around the world have drawn up clear guidelines on publishing that promote open access publishing (44, 45). For one, the EU urges European national authorities to support open access publishing (46). Similarly, in the USA, the White House Office of Science and Technology Policy presented reentry a report to the Appropriations Committees of the Senate and the House on open access publishing. That report recognized the considerable progress made by open access publishing and encouraged the federal institutions to continue efforts to implementation of open access publishing in regard to federally funded research (47).

Open access has enabled economic profit in a completely different way. Criticism has therefore been directed to these journals, as this will potentially promote quantity over quality, as the former gives better financial profit. At the same time, it also opens up the possibility to publish articles that would otherwise perhaps be difficult to publish in a traditional way due to, for example, a lack of expectation of a high degree of citation, but which will nevertheless be able to provide good innovation, either in the present or in the future. There is a broad spectrum of journals in relation to requirements for the formal quality of the articles based on the statements of the peer review. It is well known that several major discoveries and innovations were initially not understandable in the research environment of the time and were received with skepticism. It was, therefore, also challenging to publish them and spread the knowledge (16, 48, 49).

The growing popularity of open access has also led to the establishment of so-called “predatory journals,” which publish articles for financial profit, with poor or no peer review (50–52). It can be challenging to define what constitutes a predatory journal (53) and several open access journals can be liberal when it comes to accepting weaker studies for publication without this necessarily qualifying them as a predatory journal, and which may also have subsequently improved its quality. It is helpful for a researcher to check whether the journal is indexed by PubMed/MedLine or at least by Scopus and has a reasonable impact factor.

At the beginning of the 1990s so-called “preprint repositories,” where authors could post their research articles before they had been peer-reviewed and published, received acceptance in the academic society. This allowed the sharing of research broadly and freely before one had gone through the peer review process and had the article published in a journal. Expectedly, this was initially met with a certain skepticism in the research community. It is only in the last 3 years that an increasing number of serious medical journals have begun to offer posting of submitted manuscripts in preprint repositories before peer review and eventual publication as part of a more open culture around publishing. Some journals, including highly ranked journals, actually have this at present as a mandatory requirement to be able to accept manuscripts (54). There are several preprint repositories for medical research, the most well-known preprint platforms in medicine and health science are medRxiv, bioRxiv and Research Square. It is also possible to post articles in preprint repositories directly without submitting them to journals for peer review. Preprint repositories clearly warn that the content of the preprints should be used with caution because the research was not peer-reviewed and may represent erroneous conclusions and claims.

## Publication in national languages versus English

English is a dominant professional language internationally. In the last two decades, several national journals have become bilingual, duplicating the articles in English (55). The challenge with journals in the national language is that the research may have limited international impact (56, 57). At the same time, epidemiological and social medical research is focused on issues that are unique to a country, and it is often most appropriate to publish in national journals and the national language (58, 59). This is one of the elements that support the criticism against the excessive use of scientific indices such as an impact factor to measure the quality and importance of research (60). Anglicization of research has weakened the development and use of medical terminology in other languages. Many new medical terms have not received widely recognized national denotations, and doctors use English words to denote them while communicating in the national language.

Publication of the same or overlapping research in both English and the local language in different journals is controversial and can be interpreted as a double publication. Nevertheless, worldwide, there is still a large proportion of doctors, healthcare professionals, and researchers who have limited access to international journals, and many lack language skills in English. In this context, publication of the same or overlapping research in different languages may allow the availability of the research to different and broader groups of readers and more effective dissemination of knowledge (61). Reference to previous publications and agreements between the journals are required.

## Involvement in peer review and editorial work

Peer review and editorial work are important tools for developing researchers’ expertise and for quality assurance in the publication process. In contrast to editorial work, which has a visible function and is rewarded as prestigious work, peer review work is equally important but was considered a thankless job because no one could publicly quantify reviewers’ peer review effort.

The launch of Publons in 2012 has provided some incentives for peer review and editorial work (62). Publons is an internet-based tool that offers tracking, verification, and display of peer review and editorial contributions in academic journals. By the end of 2010-s, most academic journals offered crediting of peer reviews in Publons. This may have increased the motivation to undertake peer review work. Recently, Publons became part of the Web of Science – the world’s largest commercial platform that provides access to multiple databases providing reference and citation data from academic publications.

Traditionally, the peer reviews were anonymous. Since the end of the 1990s, some journals have started to publish names of peer reviewers and/or names of specific editors responsible for handling published articles. In addition, some journals give readers access to pre-publication communication history between authors, peer reviewers, and editors. This corresponds to the general trend toward openness in academia. There are both advantages and disadvantages of it. Some of these journals allow peer reviewers and editors to choose whether their names will be published and whether they allow publication of the article’s pre-publication communication.

Reviewing and editorial work is mainly based on volunteering. However, the currently observed escalating burden of reviewing and editorial work due to open access journals has raised the question of financial compensation for editorial work.

## New trends and dissemination methods

Writing and reading are the classic methods of storing and transferring knowledge. However, it is well known that a picture is worth a thousand words. An increasing degree of digitization has opened up greater use of both video and interactivity, which can convey both experimental methodology and technological innovation more easily, better, and faster (63).

Attaching explainer videos to articles has become common over the past two decades. Some journals have specialized in this. They have video publication as the primary academic product. The Video Journal of Clinical Research and the Journal of Visualized Experiments (JoVE) represent such video journals. The latter is indexed by PubMed/Medline. There are several visual academic resources related to surgery. A well-known video resource, WebSurg is not a collection of research videos but a platform with peer-reviewed educational videos on surgical techniques (64). There is a growing acceptance within academia to cite video publications (65).

A visual or graphic abstract is a visual summary of the key findings in an article. It has become common as it enables quick capture and convey of the study essence. This can be useful with an ever-increasing number of publications that can be challenging to navigate among (66). In recent years there have been demands from more and more journals to attach a visual abstract when submitting manuscripts. Visual abstract has also made it easier to present the research on social media

Just as digitization has changed everyday publishing, this has also increased the possibility and importance of using the internet and social media to disseminate research. This applies to traditional social media such as Facebook and X (earlier Twitter). Besides, there have also been established research-oriented media and forums such as Researchgate (67–70). Self-promotion via these channels is now more and more common in order to increase attention to publications and new research. There is a constantly higher social demand for the dissemination of research results to the general population.

## Artificial intelligence

The use of artificial intelligence (AI) has provided a quite new aspect of academic writing that both scientists and the publishing industry need to adapt quickly for. To approach this discussion productively, the role of AI can be grouped into various domains of publishing from idea and development to interpreting the scientific content data, to writing structure and finally in the publishing process for proofreading and journal editing (71). While AI can ease guidance through a continuous challenging and expanding scientific literature, it also points out a brand-new challenge on how original writing should be defined and the role of implementing AI-generated content. While the boundaries for both journals and academics are yet to be clearly set, several publishing guidelines already defines what’s allowed and what’s not, and how the use of AI should be disclosed when applied (72). Specific citing styles have been suggested for AI-generated text and conclusions and while most agree that pure AI-generated manuscript should not be permitted, the line between that and e.g., AI-assisted proof-reading is gray. One of the most controversial and discussed aspects is the use of AI is in Peer review. Due to the time-consuming effort of peer-review and editing along with increasing number of articles and fewer resources, AI has been argued to become a necessity to ease and comprehend the workflow of editors and reviewers (73). This could, however, certainly also undermine important innovative manuscript over more AI-friendly ones and weaken the reviewers feedback (74).
